# Nuclear expression of Survivin in paediatric ependymomas and choroid plexus tumours correlates with morphologic tumour grade

**DOI:** 10.1038/sj.bjc.6601334

**Published:** 2003-10-28

**Authors:** R A Altura, R S Olshefski, Y Jiang, D R Boué

**Affiliations:** 1 Center for Cancer Research, Columbus Children's Research Institute (CCRI), College of Medicine and Public Health, The Ohio State University, Columbus, OH, USA; 2 Department of Pediatrics, College of Medicine and Public Health, The Ohio State University, Columbus, OH, USA; 3 Center of Biopathology, CCRI, Columbus Children's Hospital and College of Medicine and Public Health, The Ohio State University, Columbus, OH, USA

**Keywords:** survivin, brain, ependyma, choroid plexus, ependymoma, choroid plexus tumour

## Abstract

Survivin is a gene that is widely expressed throughout the development of the normal mammalian embryo. Subcellular localisation of Survivin to both the nucleus and cytoplasm has suggested multiple functional roles, including inhibition of cell death, especially as demonstrated within a variety of malignant cell types, as well as regulation of the mitotic spindle checkpoint. The expression of Survivin has been associated with an adverse clinical outcome in a large number of malignancies. However, nuclear Survivin expression has been described as an independent variable of favourable prognosis in two large clinical studies of breast and gastric carcinomas. Reports of Survivin expression in normal postnatal, differentiated tissues have been restricted to cell types with high proliferative capacities, including vascular endothelium, endometrium, colonic epithelium, and activated lymphocytes. Prior to this report, expression within the normal human brain had not been characterised. Here, we analyse the expression of Survivin in human brain sections obtained from perinatal and paediatric autopsy cases. We report a strikingly high level of expression of Survivin within normal ependyma and choroid plexus (CP). Analysis of corresponding neoplastic tissue in paediatric ependymomas and CP tumours shows that expression of the nuclear form of Survivin correlates with morphologic tumour grade, with a loss of nuclear expression associated with progressive cytologic anaplasia. This pattern of expression supports a hypothesis that Survivin plays a functional role in normal ependymal growth and/or neural stem cell differentiation, and that abnormally low levels of expression of the nuclear form of this protein may be a marker of more aggressive disease and/or higher morphologic grade in ependymal and CP tumours.

Tumours of the central nervous system (CNS) are the most common subset of solid tumours presenting in children less than 15 years of age (Heideman *et al*, 1997). The overall frequency of these cancers is second only to acute lymphoblastic leukaemia (ALL) of childhood; however, the overall survival and resulting morbidity between these tumours differ tremendously. While paediatric ALL is approaching 80% long-term survival, the average survival of paediatric CNS tumours is only 45%, with the majority of children in remission suffering frequent life-long physical and neurologic deficits (Heideman *et al*, 1997). Although much work has been accomplished in differentiating paediatric CNS tumours histologically, little is still understood about their molecular abnormalities. Those abnormalities characterised to date are often distinct from those of adult brain tumours, a phenomenon that is likely related to mutational events that occur during embryologic development in paediatric tumours, within pathways essential for cell death, proliferation, and differentiation (Biegel, 1997).

Survivin is a gene with structural homology to a family of genes known as inhibitors of apoptosis (IAPs) (Li *et al*, 1998). It is widely expressed in foetal tissues and is critical for the growth and survival of the normal embryo (Uren *et al*, 2000). Genetically engineered homozygous deletion of this gene in mice results in death of the embryo early in development, between days 4 and 6 of gestation (Uren *et al*, 2000). The normal cellular function of Survivin is probably related to ensuring the accurate procession of cell division, as it is expressed in a cell-cycle-dependent manner in G2/M and has been localised to the kinetochore during the late phase of mitosis (Skoufias *et al*, 2000). Controversy exists as to the relationship between its antiapoptotic role, documented in mammalian cell culture as well as in *in vivo* tumour models, and its role as a chromosomal passenger protein in lower eukaryotes. Multiple splice forms of Survivin have been described in normal and tumour cells and Survivin has also been localised to multiple subcellular compartments within the cell, including the nucleus and cytoplasm, suggesting that it probably has more than one function (Fortugno *et al*, 2002; Krieg *et al*, 2002; Mahotka *et al*, 2002a,Mahotka *et al*, 2002b). Abnormally elevated levels of Survivin expression have been reported in a variety of adult malignancies, and *in vitro* and *in vivo* tumour studies are suggestive of Survivin playing an effective role in preventing cell death in these tumours (reviewed in Altieri, 2003). It has been proposed that Survivin represents an ideal candidate for molecular-targeted therapy in tumours expressing this gene, as its expression was originally reported to be absent in postnatal, differentiated tissues. Several recent reports, however, show evidence of Survivin expression in specific adult tissues. These have included vascular endothelial cells, colonic epithelium, normal endometrium, and lymphocytes (Konno *et al*, 2000; Gianani *et al*, 2001; Fukuda *et al*, 2002; Altieri, 2003). In addition, other investigators have shown that expression of the nuclear form of Survivin in breast and gastric carcinomas is an independent prognostic indicator of good prognosis in these tumours, suggesting that a loss of nuclear Survivin expression may play a role in tumour progression (Okada *et al*, 2001; Kennedy *et al*, 2003).

In the present study, we evaluated the protein expression of Survivin by immunohistochemistry in nonmalignant brain tissue from human perinatal and paediatric autopsy sections, and in biopsies of nonmalignant paediatric brain tissue during surgery. Interestingly, we found that normal ependymal cells and choroid plexus (CP) epithelial cells strongly express Survivin both in the nucleus and cytoplasm. We then examined the expression of Survivin in corresponding neoplastic tissue, including various well-described subtypes and grades of paediatric ependymomas and CP tumours. The expression of Survivin was detectable in the majority of tumours examined; however, the percentage of cells with distinct nuclear staining correlated strongly and inversely with morphologic tumour grade. This pattern of expression supports the hypothesis that Survivin plays a functional role in normal ependymal growth and/or neural stem cell differentiation, and that abnormally low levels of expression of the nuclear form of this protein may be a marker of disease aggression and/or morphologic grade in ependymal and CP tumours.

## MATERIALS AND METHODS

### Patient selection

Children with brain tumours of ependymal and CP origin, diagnosed and treated within the last 11 years at Columbus Children's Hospital, were selected from the pathology database, based on their pathologic diagnoses and the availability of formalin-fixed paraffin-embedded tumour material. Permission from the Children's Hospital IRB was granted for staining of slides and clinical chart review on each case. A total of 14 ependymomas (six myxopapillary (WHO grade I), five classic (WHO grade II), and three anaplastic (WHO grade III)) and nine CP tumours (three papillomas (WHO grade I), three ‘atypical’ papillomas (WHO grade I–II), and three carcinomas (WHO grade III)) were evaluated. Clinical follow-up was available on all cases, except one myxopapillary ependymoma. Treatment at diagnosis was based on histologic findings and extent of surgical resection and included surgery, radiation, and/or chemotherapy. Diagnoses were re-confirmed by clinicopathologic review by DRB for each case prior to antibody staining. The diagnosis of anaplastic ependymoma was made based on a predominance of a hypercellular, less well-differentiated component, together with increased mitotic activity (Burger, 1994). An atypical papilloma was defined by scattered mitotic activity, obvious cytologic atypia, increased nuclear : cytoplasmic ratio, and focal multilayering of the epithelium or stromal nests of the epithelium (Burger, 1994). Sections of nonmalignant brain tissue (preterm and older paediatric) obtained at autopsy and fixed within 24 h of death, as well as from a surgical case of a CP cyst from a patient without morphologic evidence of neoplastic transformation, were also analysed for Survivin expression.

### Immunohistochemistry

Blocks of nonmalignant brain tissue, obtained at autopsy from a 22-week foetus and a 5-year-old child, respectively, were formalin-fixed within 24 h of death and re-cut immediately prior to antibody staining. Diagnostic tissue blocks from a non-neoplastic CP cyst and from malignant brain tissue were reviewed for tissue integrity and degree of differentiation/atypia by the paediatric neuropathologist prior to selecting blocks for sectioning and antibody staining. Those blocks with large amounts of necrosis and/or heat or chemical injury related to either the surgical procedure or to a delay in fixation were excluded from the analysis. Four slides were cut from each nonmalignant and from each malignant block and were stained with H&E, two different polyclonal anti-Survivin antibodies, and normal rabbit serum as a negative control, respectively. Normal brain sections were inclusive of regions from the following brain areas: (1) frontal–parietal cortex and white-matter, (2) basal ganglia/periventricular white matter, (3) hippocampus/temporal cortex, (4) midbrain, (5) pons, (6) medulla, (7) cerebellum (cortex, white matter, and dentate nucleus), (8) occipital cortex, (9) thalamus/hypothalamus, and (10) spinal cord (cervical, thoracic, and lumbar).

Formalin-fixed, paraffin-embedded tissue sections were freshly cut and immunostained for Survivin using two different polyclonal anti-Survivin antibodies. One antibody, obtained from Novus Biologicals (NB-500-201 K3), was used at a dilution of 1 : 300 for each case. The other antibody, obtained from Santa Cruz (sc-10811), was used at a dilution of 1 : 400. Each antibody was pretested on tumours known to express Survivin, including human breast carcinomas and melanomas, to determine appropriate antibody dilutions. Negative controls, performed with normal rabbit Ig as the primary antibody, were used to ensure the specificity of staining.

Sections (4 *μ*m) from paraffin blocks were placed on glass slides and baked overnight at 60°C. Following passage through xylene and graded alcohols, the sections were treated with 3% hydrogen peroxide. Antigen retrieval was carried out by pressure-cooking in citrate buffer, pH 6.0. The sections were then incubated overnight with the polyclonal antibodies (or normal rabbit Ig) at 4°C. Sections were washed, and then incubated with a biotinylated secondary anti-rabbit antibody (Biocare Medical) for 20 min at room temperature. Slides were then incubated with Streptavidin HRP (Biocare Medical). After rinsing for 20 min, slides were treated with AEC (Amino-ethylcarbazole), and then counterstained with haematoxylin.

### Evaluation of immunohistochemistry results

Survivin immunoreactivity was evaluated semiquantitatively based on the percentage of cells demonstrating nuclear and/or cytoplasmic immunohistochemical staining. Scoring was performed by the paediatric neuropathologist (DRB) for each case. Staining was assessed in 5–10 high-powered fields at × 40 magnification. Slides were assigned scores of 1 if <10% positive, 2 if <10–20% positive, 3 if 21–50% positive, and 4 if >50% positive.

### Western blot

Perinatal brain tissues obtained at autopsy from a 28-week gestation age infant were frozen in dry ice, and grounded in a cold mortar containing a small amount of dry ice. The powdered brain tissue was then transferred to an Eppendorf tube. An equal weight/volume of blending buffer (containing 10% SDS, 62.5 mM Tris pH 6.8, 5 mM EDTA, 1 mM PMSF) was added and cells were further lysed using a Teflon pestle, and subsequently sonicated three times for 10 s using a Virsonic 300 sonicator (Virtis Corp., Gardiner, NY, USA) to homogenise large size DNA. Cell debris was removed by centrifugation at 14 K r.p.m. for 1 min. The protein concentration of the supernatant was determined by the BioRad DC protein assay (BioRad Laboratories, Hercules, CA, USA). Protein samples (50 *μ*g each) were separated by electrophoresis on a 12% SDS–polyacrylamide gel. The separated proteins were transferred onto a nitrocellulose membrane (Bio-Rad Laboratories, Hercules, CA, USA) using a Bio-Rad Semi-dry Transfer Cell. Survivin was detected using ECL Advance Western Blotting Detection Kit according to the manufacturer's instructions. Briefly, the membrane was blocked in 20 mM Tris-buffered saline (pH 7.6) with 0.1% Tween (TBST) containing 2% (w v^−1^) blocking powder provided by the manufacturer at 4°C overnight. The membrane was incubated with a 1 : 5000 dilution of primary antibody (sc-10811, Santa Cruz, CA, USA) for 1 h at room temperature. The membrane was then washed four times with TBST. HRP-conjugated anti-mouse secondary antibody (Santa Cruz) at a dilution of 1 : 5000 was added to the membrane and incubated at room temperature for 30 min. After four TBST washes, the blot was incubated in detection reagent for 5 min and exposed to a Hyperfilm™ECL™ film (Amersham Pharmacia Biotech, Buckinghamshire, England). Human *β*-actin, an internal control, was detected using a mouse monoclonal anti-*β*-actin antibody (Sigma, St Louis, MO, USA).

## RESULTS

### Patient characteristics

Of the 14 patients with ependymomas, the ages at diagnosis ranged from 3 to 20 years (mean=9.2 years). Myxopapillary ependymomas (six cases) were confined to the lower spinal cord (filum region) in all cases. Intracranial ependymomas were localised to the posterior fossa (six cases) and supratentorial region (two cases). Anaplastic ependymomas were located in the supratentorial region (two cases) and the posterior fossa (one case). Myxopapillary ependymomas were treated with surgical resection only (3) or resection followed by radiation (3). Treatment for intracranial ependymomas included surgical resection alone (1) or surgery followed by radiation and chemotherapy (seven cases). Follow-up ranged from 1 to 10 years after initial diagnosis. Three patients (two grade II ependymomas and one grade III ependymoma) died of disease progression within 4 years of diagnosis.

Of the nine patients with CP tumours, three had grade I papillomas, three had grade I–II (atypical) papillomas, and three had grade III choroid plexus carcinomas (CPC). Ages at diagnosis for the six patients with papillomas ranged from 4 days to 48 months (average 22.6 months). Ages at diagnosis for the patients with CPCs were 17 months, 22 months, and 14 years. Patients with grade I or grade II papillomas were treated with surgery alone. Two patients with CPC were treated with surgery, radiation, and chemotherapy. One patient with CPC died prior to initiation of therapy. Another patient with a CPC died of disease progression. The third patient with a CPC is alive, but is currently less than 1 year from diagnosis. All eight patients with CP papillomas (grade I and II) remain alive without evidence of disease progression (follow-up 2–11 years).

### Immunohistochemical analysis

**Figure 1:**
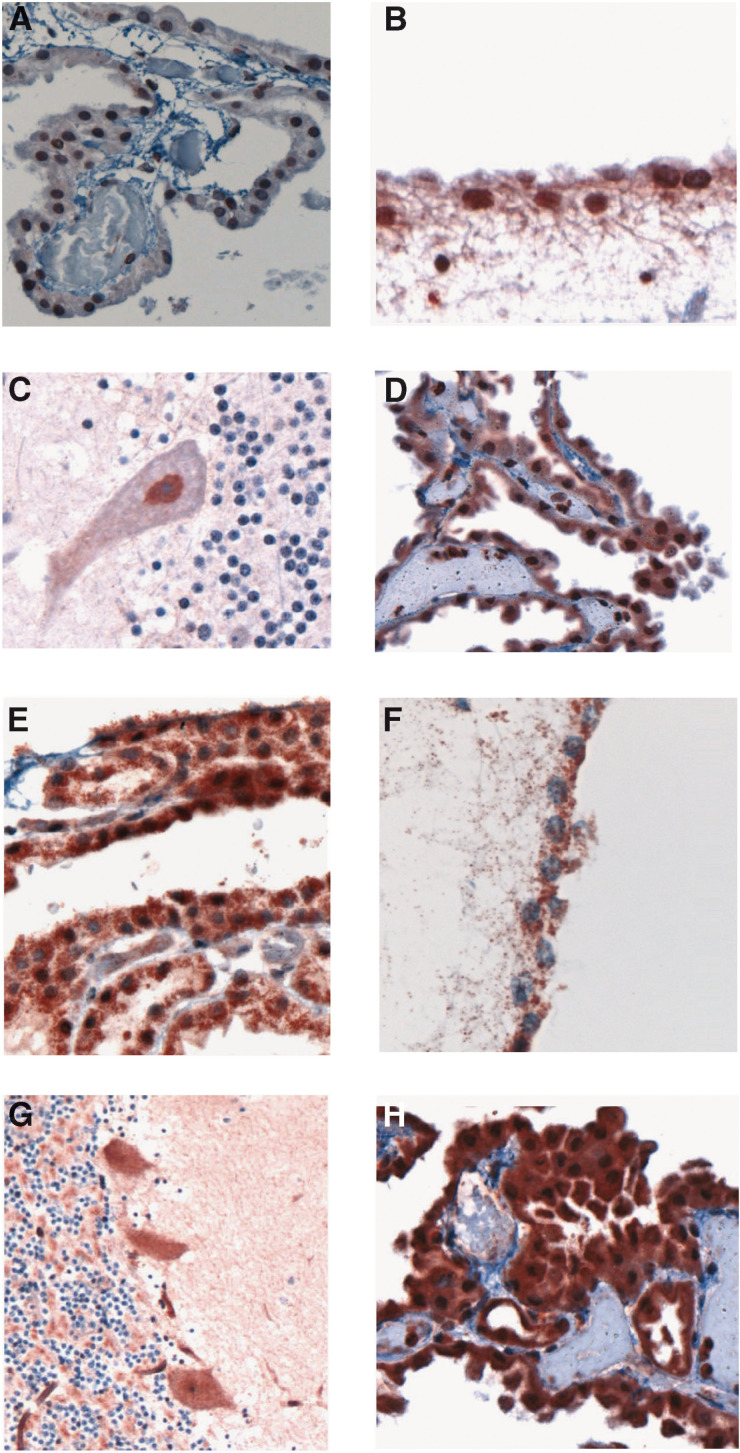
Survivin expression in normal brain tissue using the sc-10811 antisera (**A–D**) and the NB-500-201K3 antisera (**E–H**). (**A**) choroid plexus, (**B**) ependyma-fourth ventricle, (**C**) Purkinje neuron, (**D**) choroid plexus cyst; (**E**) choroid plexus, (**F**) ependyma-fourth ventricle, (**G**) Purkinje neurons, and (**H**) choroid plexus cyst. The sc-10811 antisera stain the nuclear form, while the NB-500-201K3 antisera stain the cytoplasmic form of Survivin.

Diffusely strong Survivin staining of sections from normal brain was observed in the ependymal cells lining the ventricular system, and choroidal epithelial cells comprising the CP (Figure 1

). Choroid plexus cells from a non-neoplastic CP cyst stained similarly (Figure 1D and H). Some normal neurons, including neurons within the midbrain, cerebellar dentate nucleus, Purkinje cells (Figure 1C and G), anterior horn cells of the spinal cord, and meningoepithelial cells also variably expressed Survivin. Vascular endothelial cells were diffusely positive for Survivin. Neurons within the medulla, occipital cortex, hippocampus, and thalamus were essentially negative for Survivin. Glial cells (oligodendrocytes and astrocytes) generally lacked any Survivin staining (Figure 1C and G). The pattern of Survivin staining in the ependymal, choroidal, and selective neuronal cells was predominantly cytoplasmic using the NB-2500-201K3 antibody, and was predominantly nuclear using the sc-10811 antibody (Figure 1A–D compared with E–H).

The results of immunohistochemical staining of ependymomas are shown in Table 1

**Table 1:** Survivin staining scores in ependymomas (A) and choroid plexus tumours (B)

**(A)**	**Tumour grade**	**Diagnosis**	**Age (years)**	**Nuclear Survivin staining**	**Status**
	I	Myxopapillary	16	4	Alive
	I	Myxopapillary	8	4	Unknown
	I	Myxopapillary	11	4/3	Alive, one recurrence
	I	Myxopapillary	12	4	Alive
	I	Myxopapillary	6	4	Alive
	I	Myxopapillary	14	4	Alive, drop met at dx
	II	Ependymoma	3	4	Died, progressive ds
	II	Ependymoma	6	3	Alive
	II	Ependymoma	4	3	Died, progressive ds
	II	Ependymoma	16	4	Alive
	II	Ependymoma	3	3	Alive
	III	Anaplastic	20	2	Alive, one recurrence
	III	Anaplastic	6	2	Died, progressive ds
	III	Anaplastic	4	2	Alive
					
**(B)**	**Tumour grade**	**Diagnosis**	**Age (months)**	**Nuclear Survivin staining**	**Status**
					
	I	CPP	48	4	Alive
	I	CPP	20	4	Alive
	I	CPP	4	4	Alive w/ stable ds
	I–II	ACPP	48	1	Alive
	I–II	ACPP	0.13	1	Alive
	I–II	ACPP	16	1	Alive
	III	CPC	22	1	Died, progressive ds
	III	CPC	17	1	Died, progressive ds
	III	CPC	14 year	1/2	Alive (f/u <1 year)

1=<10%; 2=10–20%; 3=21–50%; 4=>50% cells positive; CPP=choroid plexus papilloma; ACPP=atypical choroid plexus papilloma; CPC=choroid plexus carcinoma; DS=disease; DX=diagnosis.

and Figure 2

**Figure 2:**
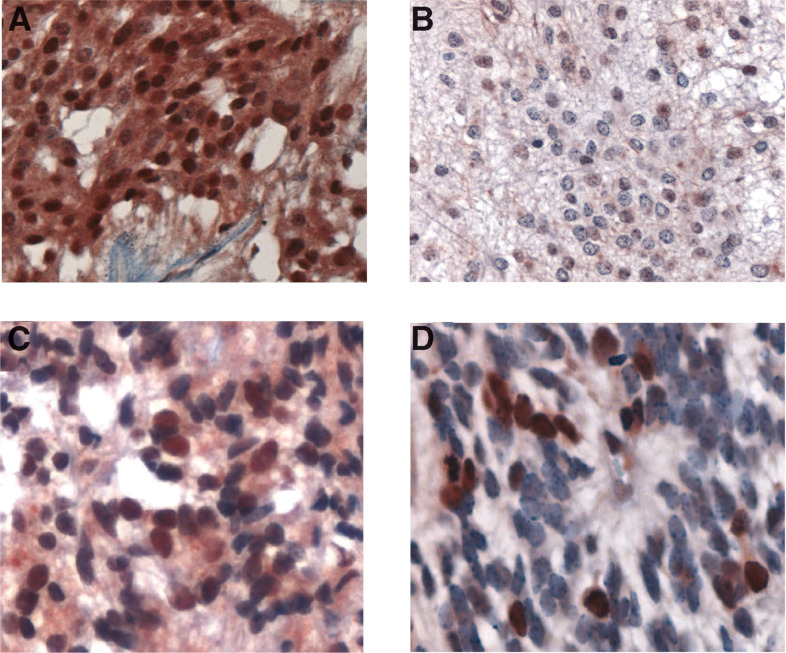
Survivin expression in ependymomas using the sc-10811 antisera: (**A**) myxopapillary ependymoma, (**B**) grade II ependymoma, (**C**) grade II ependymoma, and (**D**) anaplastic ependymoma.

. Five of six myxopapillary tumours showed between 50 and 100% of nuclear positivity for Survivin with the sc-10811 antisera. One of the six tumours had a variable pattern of expression, with some regions of tumour having greater than 90% of the nuclei positive and other regions showing only 20–30% nuclear positivity. For the grade II classic ependymomas, three of five had between 20 and 30% of the nuclei positive for Survivin and two of five had over 50% of nuclei positive. In all three grade III anaplastic ependymomas, the percent of tumour nuclei staining positive was only 10–20%.

**Figure 3:**
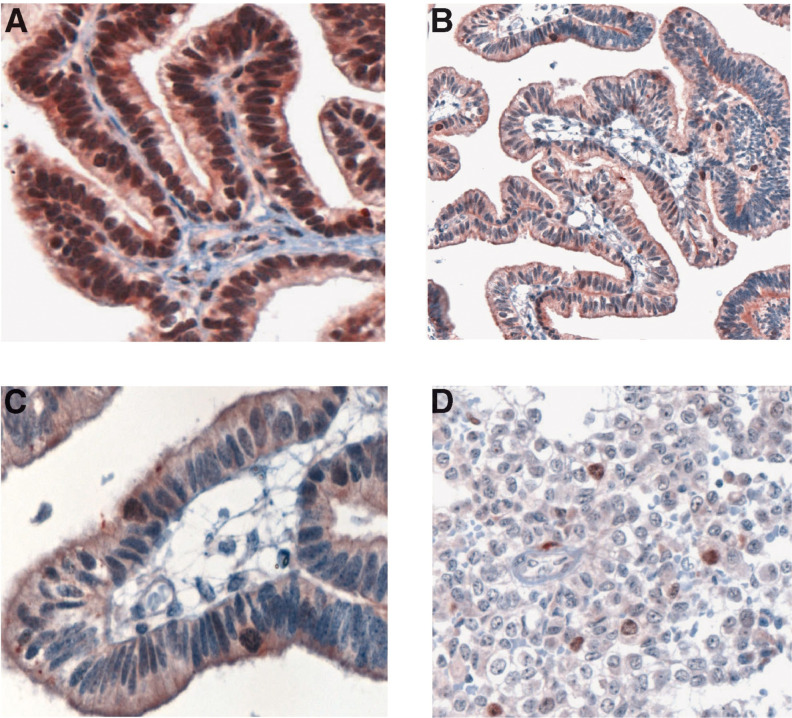
Survivin expression in choroid plexus tumours using the sc-10811 antisera: (**A**) papilloma grade I, (**B**) atypical papilloma grade I–II (low magnification), (**C**) atypical papilloma grade I–II (high magnification), and (**D**) choroid plexus carcinoma.

The results of immunohistochemical staining of CP tumours are shown in Table 1 and Figure 3

. All grade I CP tumours had 50–100% of cell nuclei positive for Survivin. By contrast, the grade I–II ‘atypical’ tumours had only 5–10% nuclear positivity and the CPC had a maximum of 10% of nuclei staining positive (generally <5%). The cells staining most intensely in both ependymal and choroid tumours were those undergoing mitosis. Cytoplasmic staining with the NB-500-201 antibody was equivalent (>50% cells) among all tumour grades. These results suggest an inverse correlation between morphologic tumour grade and nuclear Survivin staining, with higher grade tumours having less reactivity than lower grade brain tumours.

### Western blot analysis

**Figure 4:**
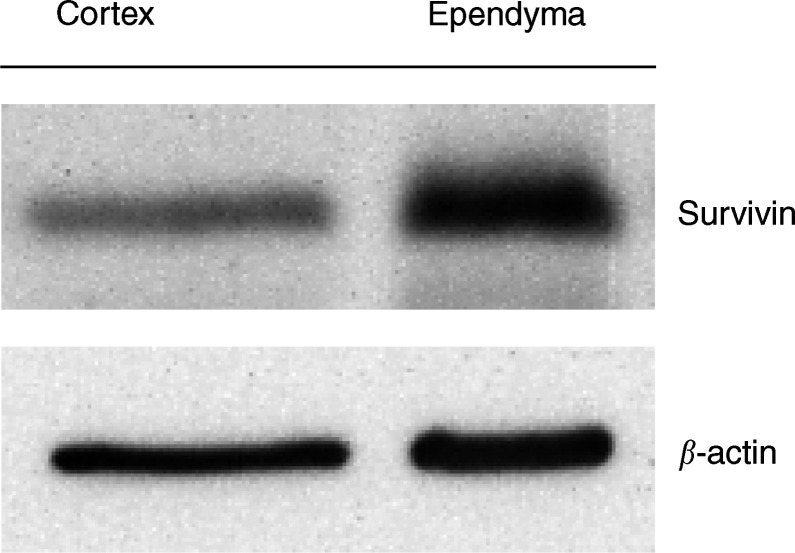
Survivin and *β*-actin protein expression in normal human ependyma and cortex.

A total of 50 *μ*g of total cellular protein, prepared from the cortex and ependyma of a 28-week gestational age infant at autopsy, was subject to SDS electrophoresis on a 12% polyacrylamide gel. Staining of blots was performed using the sc-10811 anti-Survivin antibody and a *β*-actin antibody, as described. A specific band at approximately 19 kDa, consistent with the Survivin protein, was observed. The expression of Survivin in ependymal tissue was five- to 10-fold higher than that in the cortical tissue (Figure 4

). The small amount of ‘cortical’ Survivin detected is likely derived from cortical blood vessel endothelium. These results are consistent with the immunohistochemical staining of the nonmalignant brain tissue described above, showing very high levels of Survivin in ependymal cells when compared with other areas of the brain.

## DISCUSSION

Survivin is a gene with structural homology to the inhibitor of apoptosis gene family and functions to prevent cell death in a variety of tumour subtypes (Ambrosini *et al*, 1997; Li *et al*, 1998; Altieri, 2003). It was originally reported that Survivin was normally expressed at high levels in foetal tissue, but absent in normal adult, differentiated cells (Ambrosini *et al*, 1997). Interestingly, our study of normal human brain, using two different antibodies that differentially detect nuclear and cytoplasmic Survivin protein expression, shows that both variants are present at high levels within the ependyma and CP of a 22-week foetus (essentially the time of completion of ependymal differentiation) and an older child. To our knowledge, this is the first report of Survivin expression in normal human brain tissue. Our data suggest that similar levels of Survivin expression, with a similar subcellular localisation, exists among foetal and older paediatric brain tissue. These results have many implications for understanding the functional roles of Survivin in the prenatal and postnatal development of the CNS and potentially for understanding the functional role of the ependyma, a cellular domain whose function is still incompletely understood.

In the normal adult mammalian brain, ependymal cells, composed of a single uninterrupted layer of ciliated squamous to columnar cells, comprise the lining of the cerebral ventricles and the central canal of the spinal cord (Bruni, 1998). Growth and differentiation of the ependymal lining occurs over a lengthy period of foetal development, from about the fourth week of gestation to week 22 in the human foetus (Bruni, 1998). The CP is a highly specialised type of ependymal epithelium arising after closure of the neural tube in the lateral, third, and fourth ventricles, around week 7 (Dziegielewska *et al*, 2001). The function of the ependyma is hypothesised to vary at different stages in the lifetime of the animal, based on differential protein expression analyses. Foetal ependyma plays a role in the differentiation and cellular organisation of the developing CNS, including axon guidance, motor neuron differentiation, transformation of radial glia, and nutrient transport (Bruni, 1998). Adult ependyma plays a role in regulating nutrient transport between the CSF and neuropil via the ‘blood–brain barrier’ network; however, cellular and molecular studies provide evidence that it may also be an important region for the generation of new neurons (Morshead and van der Kooy, 2001). The subgranular layer of the hippocampal dentate gyrus and the forebrain periventricular region (PVR) are the only two neurogenic regions that have been described in the adult mammalian brain (Alvarez-Buylla *et al*, 2001). Controversy still exists as to the exact cellular origin of the neural stem cell in the PVR; however, cell–cell interactions within the subventricular zone and the ependymal layer are likely critical for the growth and maintenance of these cells (Alvarez-Buylla *et al*, 2001). The expression of Survivin within the ependyma of normal human brain, as shown by our group, is intriguing in the context of a region that might be critical for the support of neural stem cell growth. As a genetic disruption of Survivin directed exclusively to neural tissue has not yet been reported, it is unknown as to what effect the absence of this protein would incur on normal neural development or in the context of neuronal injury.

Reports of Survivin expression in CNS tumours include two studies of gliomas and astrocytomas (Chakravarti *et al*, 2002; Kajiwara *et al*, 2003), and one study of mixed subtypes of CNS tumours (Sasaki *et al*, 2002). The former two studies showed a correlation between high levels of Survivin and higher grade tumours, as assayed by Western blot and reverse transcriptase polymerase chain reaction (RT–PCR). In the latter study of mixed CNS subtypes, the authors were unable to demonstrate a correlation between Survivin expression, as assayed by IHC, and tumour grade. In retrospect, all these studies used reagents that would predominantly detect the cytoplasmic form of Survivin. The antibodies used in two of the studies detected cytoplasmic forms of the protein, and the primers used for the RT–PCR study detected only one isoform of Survivin known to be cytoplasmic. Analysis of Survivin protein expression in our study of paediatric ependymal and CP tumours showed that Survivin is expressed in all morphologic grades of these tumours. Using two different Survivin antisera, we were able to detect predominantly nuclear and predominantly cytoplasmic forms of Survivin, respectively. Although both antibodies had some overlapping reactivity, each stained these subcellular regions with varying opposing intensity. Interestingly, the percent of nuclei staining positive using the sc-10811 antisera was lower in morphologically higher grade, less differentiated brain tumours (both ependymal and CP), than the percent staining positive in the lower grade tumours. This implies, functionally, that the selective loss of expression of the nuclear form of Survivin may play a role in tumour progression or anaplasia. These data are consistent with that of recent publications of Survivin expression in breast and gastric carcinomas, which demonstrated that lower nuclear expression of Survivin was an independent prognostic indicator of poor prognosis (Okada *et al*, 2001; Kennedy *et al*, 2003). As the number of cases in our study was not large enough to assess the impact of nuclear Survivin staining as an independent prognostic factor in this subset of brain tumours, we cannot defend its use as a marker for clinical outcome at this time. Our study does support a potential use for the sc-10811 antibody in determining or helping to confirm the morphologic grades of ependymomas and choroidal tumours, as these particular brain tumours are notoriously difficult to grade on the basis of histology alone (Burger, 1994). Our data are not inconsistent with the findings of other studies that identify high cytoplasmic expression of Survivin as a prognostic factor in therapeutically resistant tumours, as results here using the NB-500-201 antibody show that high levels of cytoplasmic Survivin are expressed in higher grade ependymomas and CPC. The percent of cells with cytoplasmic staining, however, did not correlate with tumour grade in our study, consistent with the results in CNS tumours described previously (Sasaki *et al*, 2002).

**Figure 5:**
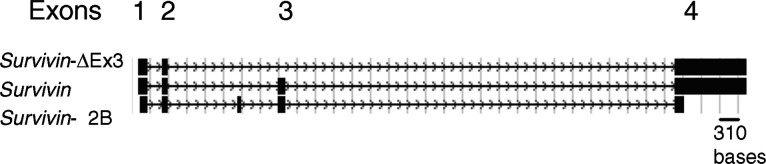
Genomic structure of the three known isoforms of *Survivin: Survivin*-ΔEx3, *Survivin*, and *Survivin*-2B (adapted from The UCSC Genome Browser Database).

As discussed in detail in a recent publication of Survivin expression in breast carcinomas, as well as in several intracellular localisation studies of Survivin protein, multiple pools of Survivin exist within transformed cells and these likely have different functions (Fortugno *et al*, 2002; Mahotka *et al*, 2002b; Kennedy *et al*, 2003). Several different splice variants of Survivin also exist (Survivin, Survivin delta Ex 3, and Survivin-2B), as illustrated in Figure 5

. These have been shown to be transcribed at variable levels within tumour cells and to have varying degrees of antiapoptotic activity (Mahotka *et al*, 2002a,Mahotka *et al*, 2002b). Intracellular localisation of the Survivin isoforms identified that the delta Ex 3 mutant localises exclusively to the nucleus and that Survivin and Survivin-2B localise to the cytoplasm (Mahotka *et al*, 2002b; Rodriguez *et al*, 2002). The delta Ex3 isoform has a novel C-terminal amino-acid sequence within which resides a nuclear localisation signal (NLS) (Mahotka *et al*, 2002b; Rodriguez *et al*, 2002). Interestingly, mutation studies of Survivin identified that its cytoplasmic localisation results from its active export from the nucleus and imply that the biological characteristics of Survivin itself within tumour cells might account for the clinical properties of the tumour (Rodriguez *et al*, 2002). Cytoplasmic Survivin has been characterised as antiapoptotic, as requiring post-translational modification by phosphorylation of a threonine residue for apoptotic inhibition, and as associating with microtubules (O'Connor *et al*, 2002). Nuclear Survivin has been proposed to function as a kinetochore-associated, chromosomal passenger protein, which may serve a role in maintaining the integrity of the mitotic spindle (Uren *et al*, 2000). Disruption of this latter function, either through the altered accumulation of the delta Ex3 form of Survivin or through an aberrant mechanism of Survivin export from the nucleus, has huge implications for its role in cancer and tumour progression, including the development of aneuploidy and the accumulation of new genetic mutations.

In summary, in this work we have identified Survivin expression in normal ependymal and choroidal brain cells as well as in their corresponding neoplastic counterparts, and have suggested a role for the quantitation of nuclear Survivin expression in the grading of ependymal and choroidal neoplasia. Larger studies examining the role of Survivin as an independent prognostic factor and its functional role in these tumours as well as in normal brain tissue during development and in response to injury need to be undertaken.
